# Sneathia vaginalis and Sneathia sanguinegens elicit conserved inflammatory responses in a human 3D cervical cell model

**DOI:** 10.1099/mic.0.001709

**Published:** 2026-05-21

**Authors:** Paweł Łaniewski, Anita Garwolinska, Gwenllian Anwyl, Melissa M. Herbst-Kralovetz

**Affiliations:** 1 Department of Basic Medical Sciences, College of Medicine-Phoenix, University of Arizona, Phoenix, AZ, USA; 2 Department of Life Sciences, University of Bath, Bath, UK; 3 Department of Obstetrics and Gynecology, College of Medicine-Phoenix, University of Arizona, Phoenix, AZ, USA

**Keywords:** bacterial vaginosis, carcinogenesis, host defense, inflammation, urethritis, vaginal microbiota

## Abstract

*Sneathia* species are emerging pathogens associated with adverse gynaecologic, obstetric and neonatal sequelae, including bacterial vaginosis, preterm birth, chorioamnionitis, non-gonococcal urethritis and HPV-mediated cervical carcinogenesis. Yet, their pathogenicity is poorly understood due to their fastidious nature. Here, we conducted infection assays using multiple strains of *Sneathia vaginalis* and *Sneathia sanguinegens* to evaluate host responses in an *in vitro* 3D organotypic model of the human cervix. We profiled a broad array of proteins, including antimicrobial peptides, cytokines, growth factors, mucins, matrix metalloproteinases and proteins related to apoptosis and cellular stress. Both *Sneathia* species induced similar pro-inflammatory signatures. Notably, all strains robustly induced IL-6 and IL-8/CXCL8 (*P* from 0.02 to <0.0001), which are clinical markers associated with preterm birth, vaginal dysbiosis and cervical carcinogenesis. Most strains also induced the two chemokines IP-10/CXCL10 and MIP-3*α*/CCL20. Conversely, no significant induction of matrix metalloproteinases or apoptosis-related proteins was observed. Furthermore, co-infection with both species elicited similar cytokine patterns with no synergistic effects observed. Collectively, investigation of multiple strains confirms that both *Sneathia* species trigger a conserved immune-mediated response. This reveals a shared pathogenic mechanism that represents a potential pathway toward adverse gynaecologic and obstetric outcomes.

## Data Availability

All data supporting this study are included in the article and its supplementary files. Further inquiries should be directed to the corresponding author.

## Introduction

The clinical significance of *Sneathia* species has long been underappreciated. Due to complex nutritional and atmospheric requirements, these anaerobes were often missed by standard hospital cultures. However, the advent of molecular techniques for microbial identification has revealed associations of *Sneathia* with multiple adverse gynaecologic, obstetric and neonatal health outcomes [1].

*Sneathia* commonly colonizes the vagina and cervix; in one study, it appeared in over 40% of vaginal samples [2]. Two species have been identified: *Sneathia vaginalis* and *Sneathia sanguinegens* [3]. *S. vaginalis* is more prevalent than *S. sanguinegens*, yet they often co-occur within the vaginal microbiome [2]. Both species are implicated in bacterial vaginosis (BV), which affects approximately one-third of reproductive-age women. Interestingly, *Sneathia* spp. exhibit a higher prevalence in patients presenting with symptoms [4]. Accordingly, recent molecular diagnostics for BV incorporate *Sneathia* as a key indicator of the condition within multi-species panels [5].

Notably, *Sneathia* can disseminate to distant organs or become systemic. Both species have been isolated in cases of peri- and postpartum bacteraemia and linked to preterm premature rupture of membranes (PPROM), preterm birth, chorioamnionitis, neonatal meningitis and miscarriage [1]. *Sneathia* has also been implicated in sexually transmitted infections (STIs), including human papillomavirus (HPV). Multiple studies have reported increased *Sneathia* abundance in individuals diagnosed with HPV and cervical neoplasia [6], suggesting these bacteria might contribute to viral persistence and disease progression [7].

Despite increasing evidence of their clinical relevance, the mechanisms by which *Sneathia* spp. facilitate a harmful microenvironment, which can lead to severe adverse health outcomes, remain poorly understood. Here, we determined host responses to *S. vaginalis* and *S. sanguinegens in vitro* utilising multiple clinically relevant bacterial isolates and an organotypic human three-dimensional (3D) cervical epithelial cell model to better understand the mechanistic contributions of these two *Sneathia* species to obstetric and gynaecologic pathologies.

## Methods

### Bacterial strains

*Sneathia* strains used in the study are listed in Table 1. Bacteria were cultured on brain heart infusion agar (Beckton, Dickinson and Company) supplemented with 5% human serum, 1% yeast extract, 2% gelatine, 0.1% starch and 0.1% glucose [2]. Bacterial cultures were maintained at 37 °C under anaerobic conditions generated using an AnaeroPack System (Mitsubishi Gas Chemical).

**Table 1.** Bacterial strains used in this study. Three *S. vaginalis* strains and two *S. sanguinegens* strains, including the type strain of each species, were obtained from the Biodefense and Emerging Infections (BEI) Research Resources Repository or the Culture Collection University of Gothenburg (CCUG)

Species	Strain	Alternative ID	Characteristic	Source
*S. vaginalis*	SN35	Sn35	Isolated from the vagina of a woman presenting with symptoms of preterm labour at 26 weeks of gestation; Richmond, VA, USA (2011)	BEI
	CCUG 52976	DSM 16630, T 3315/96	Isolated from the blood of a 36-year-old woman with peripartum bacteraemia; Strasbourg, France (2006)	CCUG
	CCUG 52977^T^	DSM 16631, T 1490/99	Type strain; isolated from the blood of a 32-year-old woman with peripartum bacteraemia; Strasbourg, France (2006)	CCUG
*S. sanguinegens*	CCUG 41628^T^	CIP 106906	Type strain; isolated from the blood of a 32-year-old woman with complicated delivery; Örebro, Sweden (1999)	CCUG
	CCUG 52978	DSM 16650; T 31378/97	Isolated from the blood of a 21-year-old woman with peripartum bacteraemia; Strasbourg, France (2006)	CCUG

### Human 3D cervical epithelial cell model

A human 3D cervical epithelial cell model was generated as previously described [8,9]. Briefly, human endocervical epithelial cells (A2EN) were cultured on collagen-coated dextran microcarrier Cytodex-3 beads (Cytiva) in rotating wall vessel bioreactors (Synthecon) at 37 °C in a humidified atmosphere of 5% carbon dioxide. The bioreactors were rotated at 20 r.p.m., and the supplemented keratinocyte serum-free medium (Gibco) was changed daily. After 28 days of culture, 3D cell aggregates were tested for viability and seeded into 24-well plates (averaging 3.7×10^5^ cells ml^−1^) for experimental manipulations.

### Infection assays

Two-day bacterial cultures were resuspended in sterile PBS and adjusted to an optical density at 600 nm (OD_600_) of 0.5. Human 3D cervical cell cultures were infected at a multiplicity of infection (MOI) of ~50 (25 μl of bacterial inoculum per 1×10^5^ cervical cells). For co-infections, equal parts of *S. vaginalis* and *S. sanguinegens* were mixed to a total OD_600_ of 0.5. Dose-response assays utilised MOIs of ~10, ~50 and ~100. Preliminary dose- and time-response assays identified these MOIs and the 24 h timepoint as optimal for studying host responses to these specific species. Controls included a sterile PBS treatment and heat-inactivated bacteria (65 °C, 15 min; MOI 100). Infected cell cultures were incubated for 24 h at 37 °C. Following 24 h incubation at 37 °C under anaerobic conditions, 3D cell aggregates and cell culture supernatants were harvested. Cytotoxicity was evaluated via the Pierce LDH Cytotoxicity Assay, and remaining samples were stored at –80 °C.

### Quantification of soluble proteins

Cell culture supernatants were analysed for 25 soluble proteins. Levels of HSP70, IL-1*α*, IL-1*β*, IL-1Ra, IL-6, IL-8/CXCL8, IL-18, IP-10/CXCL10, MCP-1/CCL2, MCP-3/CCL7, MIG/CXCL9, MIP-1*α*/CCL3, MIP-1*β*/CCL4, MIP-3*α*/CCL20, MMP-1, MMP-7, MMP-9, MMP-10, RANTES/CCL5, sFas, TGF-*α*, TRAIL and VEGF-A were determined using MILLIPLEX multi-analyte panels cytometric bead arrays (Millipore). Data were collected with a Bio-Plex 200 instrument and analysed using Bio-Plex Manager 5.0 software (Bio-Rad). Additionally, HE4/WFDC2 and SLPI/WFDC4 levels were measured using DuoSet ELISA (Bio-Techne) and an Infinite M Plex microplate reader (Tecan). All samples were assayed in duplicate. Protein concentrations were calculated using a five-parameter logistic regression and log-transformed for statistical analysis.

### Mucin gene expression

Total RNA was extracted from 3D cell aggregates (Quick-RNA Plus Kit, Zymo Research), and 1 µg was used for cDNA synthesis (iScript cDNA Synthesis Kit, Bio-Rad). Gene expression of mucins (*MUC1, MUC4*, *MUC5AC* and *MUC16*) was analysed by quantitative real-time PCR (qPCR) using iTaq Universal SYBR Green Supermix (Bio-Rad) and a QuantStudio 6 Flex System (Applied Biosystems) with primers listed in Table S1 (available in the online Supplementary Material). Expression levels were normalised to *GAPDH*, and fold changes relative to PBS controls were calculated using the 2^–∆∆Ct^ method.

### Statistical analyses

Experiments were performed using three to six independent biological replicates. Differences in cytotoxicity, protein concentrations and transcript levels (∆C_t_) were evaluated using a mixed-effects model, with infection as a fixed effect and biological replicate as a random effect. Significant results (*P*<0.05) were followed by Dunnett’s or Tukey’s post-hoc tests for multiple comparison adjustments. All analyses were conducted using Prism 10 (GraphPad).

## Results

We utilised three *S. vaginalis* and two *S. sanguinegens* strains, including two type strains (Table 1). These strains were originally isolated in Europe or the USA between 1999 and 2011 from human clinical samples. Four isolates had been cultivated from blood cultures of women with peripartum bacteraemia or complicated delivery, while one isolate had been obtained from a vaginal swab of a woman presenting with preterm labour. To evaluate the impact of these clinically relevant isolates on host defence responses, we utilised our well-characterized organotypic human 3D cervical epithelial cell model.

Initially, we infected 3D cervical cell aggregates with individual bacterial strains for 24 h. Following infection, no significant cytotoxicity was observed in infected cells (*P*>0.05) compared to the PBS-treated controls (Fig. S1). To comprehensively evaluate host responses, we profiled a broad array of proteins secreted by the cervical epithelial cells. This included 17 cytokines and growth factors, 2 proteins related to cellular stress or apoptosis, 4 matrix metalloproteinases (MMPs) and 2 antimicrobial peptides (AMPs) (Figs S2 and S3). Moreover, we evaluated the gene expression of the four most abundant mucins in our 3D cervical model (Fig. S4).

**Fig. 1.**
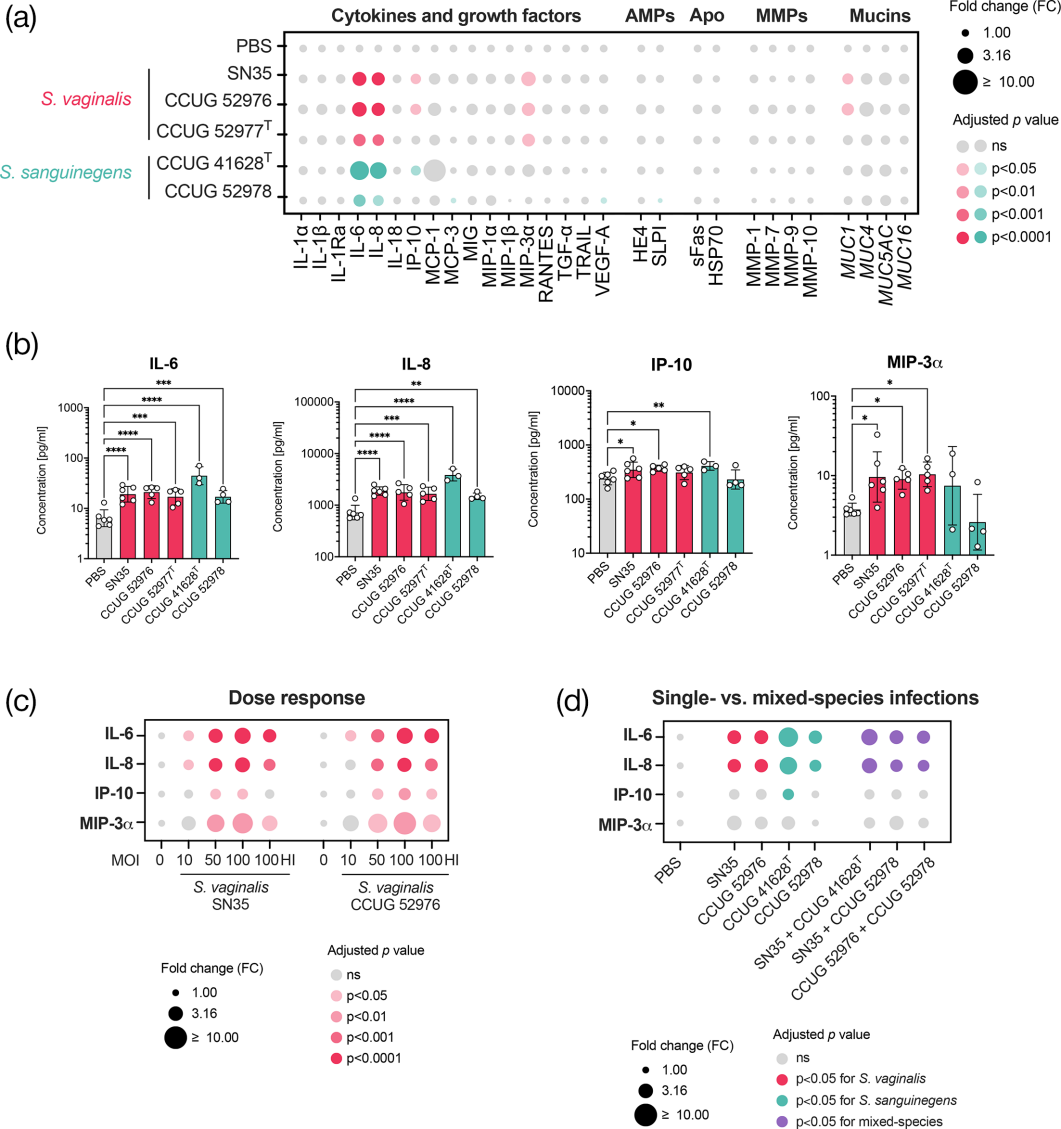
Both *S. vaginalis* and *S. sanguinegens* induce secretion of key genital inflammatory immune mediators in a human 3D cervical epithelial cell model in a dose-dependent manner. (**a**) The bubble plot shows relative levels of cytokines, growth factors, AMPs, cellular stress and apoptosis-related proteins (Apo), MMPs and mucins in 3D cell culture supernatants following infection with three individual *S. vaginalis* strains (indicated in red) and two *S. sanguinegens* strains (indicated in green) for 24 h under anaerobic conditions. (**b**) The bar charts illustrate the concentrations of key immune mediators, IL-6, IL-8, IP-10/CXCL10 and MIP-3*ɑ*/CCL20, that were altered in the 3D cell model by at least three tested strains. (**c**) The bubble plot displays fold change differences in mean levels of IL-6, IL-8, IP-10/CXCL10, MIP-3*ɑ*/CCL20 in 3D cell culture supernatants following infection with *S. vaginalis* strain SN35 and CCUG 52976 at MOI of ~10, ~50 and ~100 relative to uninfected PBS controls. Heat-inactivated bacteria at an MOI of ~100 were used as additional controls. (**d**) The bubble plot shows fold change differences in mean levels of IL-6, IL-8, IP-10/CXCL10 and MIP-3*ɑ*/CCL20 in 3D cell culture supernatants following co-infection with *S. vaginalis* and *S. sanguinegens* strains (indicated in purple) compared to infections with individual strains and PBS controls. Statistical analysis was performed using a mixed-effects model with Dunnett’s correction for multiple comparisons. ns, not significant. *P<0.05, **P<0.01, ***P<0.001, ****P<0.0001 (indicated by coloured bubbles).

Overall patterns of secreted proteins were consistent across the tested *Sneathia* strains and species, with prominent induction of IL-6 and IL-8/CXCL8 (Fig. 1a). All tested *S. vaginalis* and *S. sanguinegens* strains significantly increased levels of IL-6 (*P* from 0.0007 to <0.0001) and IL-8/CXCL8 (*P* from 0.0021 to <0.0001) relative to PBS controls. Additionally, most strains induced two other cytokines; IP-10/CXCL10 was significantly elevated by two *S. vaginalis* strains (*P*=0.03 and 0.05) and one *S. sanguinegens* strain (*P*=0.0021), while MIP-3*α*/CCL20 was significantly increased by all three *S. vaginalis* strains (*P*=0.03–0.01) (Fig. 1b). Furthermore, we demonstrated that the secretion of these key immune mediators is dose-dependent following infection with two *S. vaginalis* strains (Fig. 1c). Notably, the inactivation of bacteria at the highest dose attenuated the secretion of these mediators, although levels remained significantly higher (*P*<0.05) than those of the PBS controls (Fig. S5).

No significant induction of sFas, HSP-70, MMPs or HE4/WFDC2 was observed following infection with any of the tested strains (Fig. S3). SLPI/WFDC4 levels were significantly (*P*=0.02) decreased following infection with one *S. sanguinegens* strain. Regarding mucins, all three *S. vaginalis* strains significantly (*P*=0.04–0.02) induced *MUC1* expression relative to PBS controls; however, only one strain met the twofold threshold for biological significance (Fig. S4).

Since *S. vaginalis* and *S. sanguinegens* frequently co-occur [2], we also co-infected our 3D cervical cell aggregates. For these dual-species infection assays, we utilised three different combinations of *S. vaginalis* and *S. sanguinegens* strains. Co-infections resulted in cytokine patterns similar to those observed with single-species infections (Fig. 1d). We observed significant elevations of IL-6 (*P* from 0.004 to <0.0001) and IL-8 (*P* from 0.02 to <0.0001) compared to PBS controls (Fig. S6), suggesting no synergistic effect of co-infection on the host response.

## Discussion

We demonstrated that both *S. vaginalis* and *S. sanguinegens* induce a conserved immune-mediated response *in vitro* using a human 3D cervical epithelial model. All tested strains were potent inducers of IL-6, a pleiotropic proinflammatory cytokine, and IL-8, a chemokine that recruits neutrophils to the site of infection. Notably, these two cytokines have been implicated in preterm birth and extensively evaluated in amniotic fluid as clinical biomarkers for this condition [10]. IL-6 can promote collagen breakdown and induce the production of MMPs and prostaglandins, subsequently triggering a cascade of events: cervical ripening, PPROM and uterine contractions. During preterm labour, IL-6 can originate from intraamniotic or systemic infections, both of which have been linked to the ascent or dissemination of *Sneathia* spp. from the lower reproductive tract [1]. IL-8, induced by *Sneathia* spp., may further contribute to cervical ripening and PPROM by recruiting neutrophils, which release MMPs and other proteases [11]. Interestingly, we did not observe significant secretion of MMPs by epithelial cells in our 3D model, suggesting cell-type-specific induction of MMPs following bacterial or immune stimuli. Overall, the inflammatory potential of *S. vaginalis* and *S. sanguinegens* may increase the risk of preterm delivery; consequently, these bacterial species could serve as microbial markers for risk stratification.

HPV-infected keratinocytes also overexpress IL-6 [12]. This induction of IL-6 contributes to a chronic inflammatory state and a highly proliferative microenvironment via the STAT3 signalling pathway, while simultaneously suppressing antiviral Th1 responses. Thus, additional IL-6 production resulting from the presence of *Sneathia* in the cervix may create a microenvironment that favours HPV infection, persistence and progression to intraepithelial lesions and cancer. IL-8 signalling in the context of HPV is complex, as the virus initially suppresses its expression to evade immune detection and establish persistent infection. Conversely, the induction of IL-8 by *Sneathia* spp. might contribute to later stages of cervical carcinogenesis [13]; high IL-8 levels promote angiogenesis via CXCR1 and CXCR2 receptors (independently of VEGF), metastasis through epithelial-to-mesenchymal transition and cell proliferation by activating the ERK signalling pathway. This illustrates a complex interplay between HPV and *Sneathia* in modulating immune activity, which can facilitate viral infection and promote neoplastic disease.

IL-6 and IL-8 are also frequently elevated in the vaginal washes of women with BV [14]. Our findings suggest that *Sneathia* spp. – common constituents of polymicrobial BV biofilms [4] – may contribute to the local increase in these cytokines *in vivo*. Specific *Sneathia* strains also induced MIP-3*α*, a chemoattractant for lymphocytes and dendritic cells, as well as IP-10, which recruits activated T cells, NK cells and monocytes to the site of infection. This enhanced immune activation can increase the risk of acquiring and transmitting STIs, including HIV. Despite elevated cytokine levels, BV is not characterized by overt tissue inflammation or excessive neutrophil infiltration. Thus, certain BV-associated bacteria (e.g. *Gardnerella*) likely evade immune responses by utilising biofilm barriers, enzymatically degrading chemokines or inhibiting chemotaxis via metabolites [15].

Collectively, this study demonstrates that both *S. vaginalis* and *S. sanguinegens* possess significant pathogenic potential. The identification of a shared immune-mediated mechanism offers a biological basis for further investigating the clinical risks associated with these emerging pathogens, most notably their roles in BV, preterm birth and HPV-mediated carcinogenesis.

## Supplementary material

10.1099/mic.0.001709 Supplementary Material 1.

## Abbreviations

AMP: antimicrobial peptide
BV: bacterial vaginosis
3D: three-dimensional
HPV: human papillomavirus
MMP: matrix metalloproteinase
MOI: multiplicity of infection
PPROM: preterm premature rupture of membranes
qPCR: quantitative real-time PCR
STIs: sexually transmitted infections
