# Supplementary material for: Sneathia vaginalis and Sneathia sanguinegens elicit conserved inflammatory responses in a human 3D cervical cell model
Source: Microbiology (Reading). 2026 May 21;172(5):001709. doi: 10.1099/mic.0.001709 (PMC13193622; doi:10.1099/mic.0.001709)
Supplement: Supplementary Material 1. [file mic-172-01709-s001.pdf]

SUPPLEMENTARY MATERIAL

Łaniewski et al. *Sneathia vaginalis* and *Sneathia sanguinegens* Elicit Conserved Inflammatory Responses in a Human 3D Cervical Cell Model.

Supplementary Table S1. Primers used in this study.

Gene	Forward primer sequence	Reverse primer sequence	Ref.
GAPDH	5'-TCA TGA CCA CAG TCC ATG CCA-3'	5'-CCC TGT TGC TGT AGC CAA ATT-3'	[1]
MUC1	5'-TGC CTT GGC TGT CTG TCA GT-3'	5'-GTA GGT ATC CCG GGC TGG AA-3'	[2]
MUC4	5'-CAG CCT CTG CCA GCA CCT CAC CTG-3'	5'GGA GGG GTT TGA TGA AAA CCT TGT CGT CTC TCC-3'	[3]
MUC5AC	5'-TCCGAGGCCACCTGTGAGGG-3'	5'-GAC ATC TCG GAG CAG GAA GC-3'	[3]
MUC16	5'-GCC TCT ACC TTA ACG GTT ACA ATG AA-3'	5'-GGT ACC CCA TGG CTG TTG TG--3	[4]

[1] Gardner JK, et al. Interleukin-36γ is elevated in cervicovaginal epithelial cells in women with bacterial vaginosis and in vitro after infection with microbes associated with bacterial vaginosis. J Infect Dis. 2020; 221(6):983-988.

[2] Al-Azemi M, et al. The expression of MUC1 in human Fallopian tube during the menstrual cycle and in ectopic pregnancy. Hum Reprod 2009; 24:2582-2587.

[3] Gipson IK, et al. MUC4 and MUC5B transcripts are the prevalent mucin messenger ribonucleic acids of the human endocervix. Biol Reprod 1999; 60:58-64.

[4] Russo CL, et al. Mucin gene expression in human male urogenital tract epithelia. Hum Reprod 2006; 21:2783-2793.

Supplementary Figures

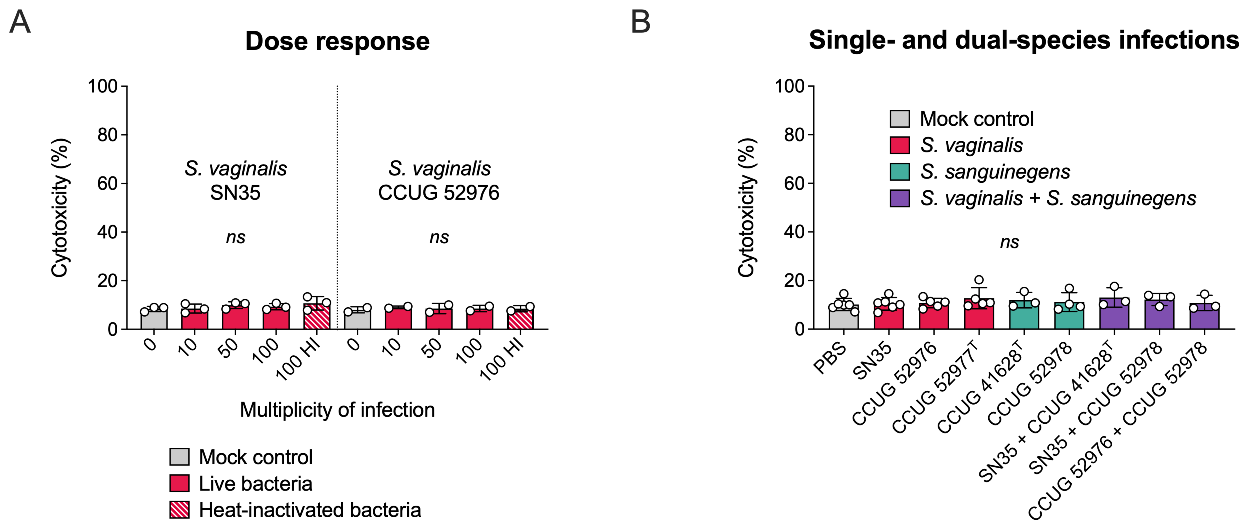

Supplementary Figure S1. Cytotoxicity in human 3D cervical epithelial cell models following infection with *Sneathia* species.

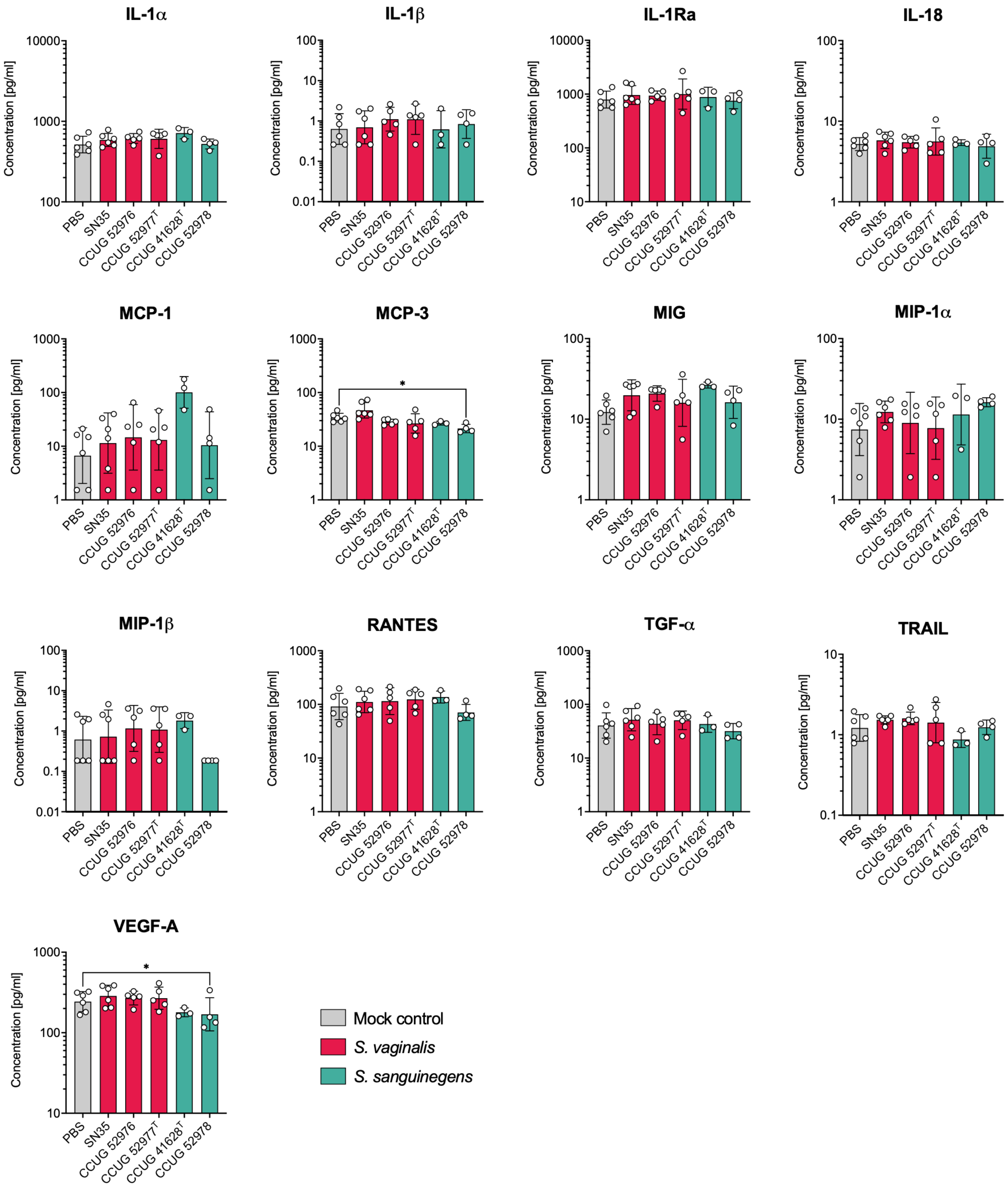

Supplementary Figure S2. Secretion of cytokines and growth factors in a human 3D cervical epithelial cell model following infection with *S. vaginalis* and *S. sanguinegens* strains.

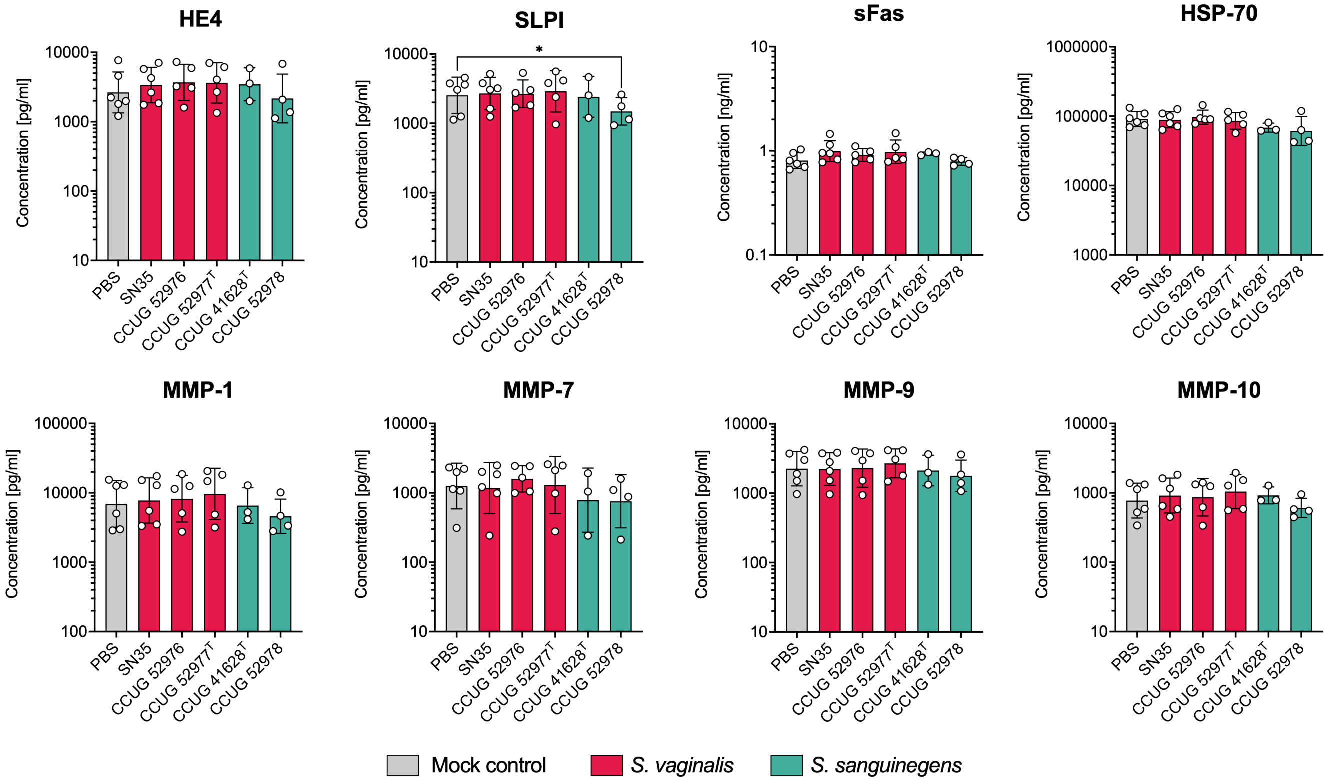

Supplementary Figure S3. Secretion of apoptosis-related proteins and matrix metalloproteinases (MMPs) in a human 3D cervical epithelial cell model following infection with *S. vaginalis* and *S. sanguinegens* strains.

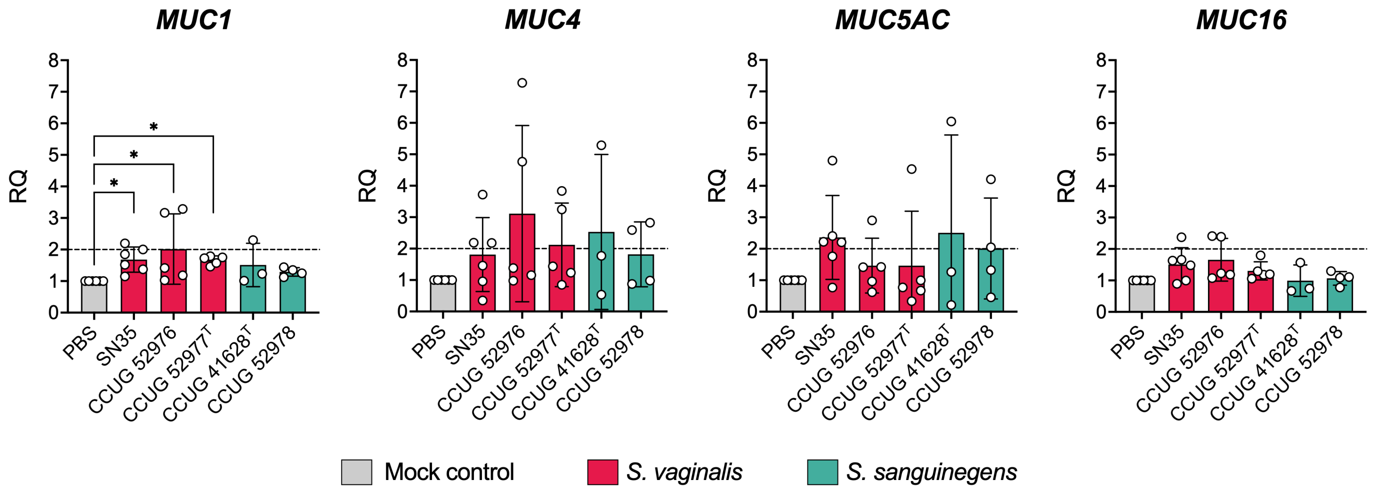

Supplementary Figure S4. Expression of mucins in a human 3D cervical epithelial cell model following infection with *S. vaginalis* and *S. sanguinegens* strains.

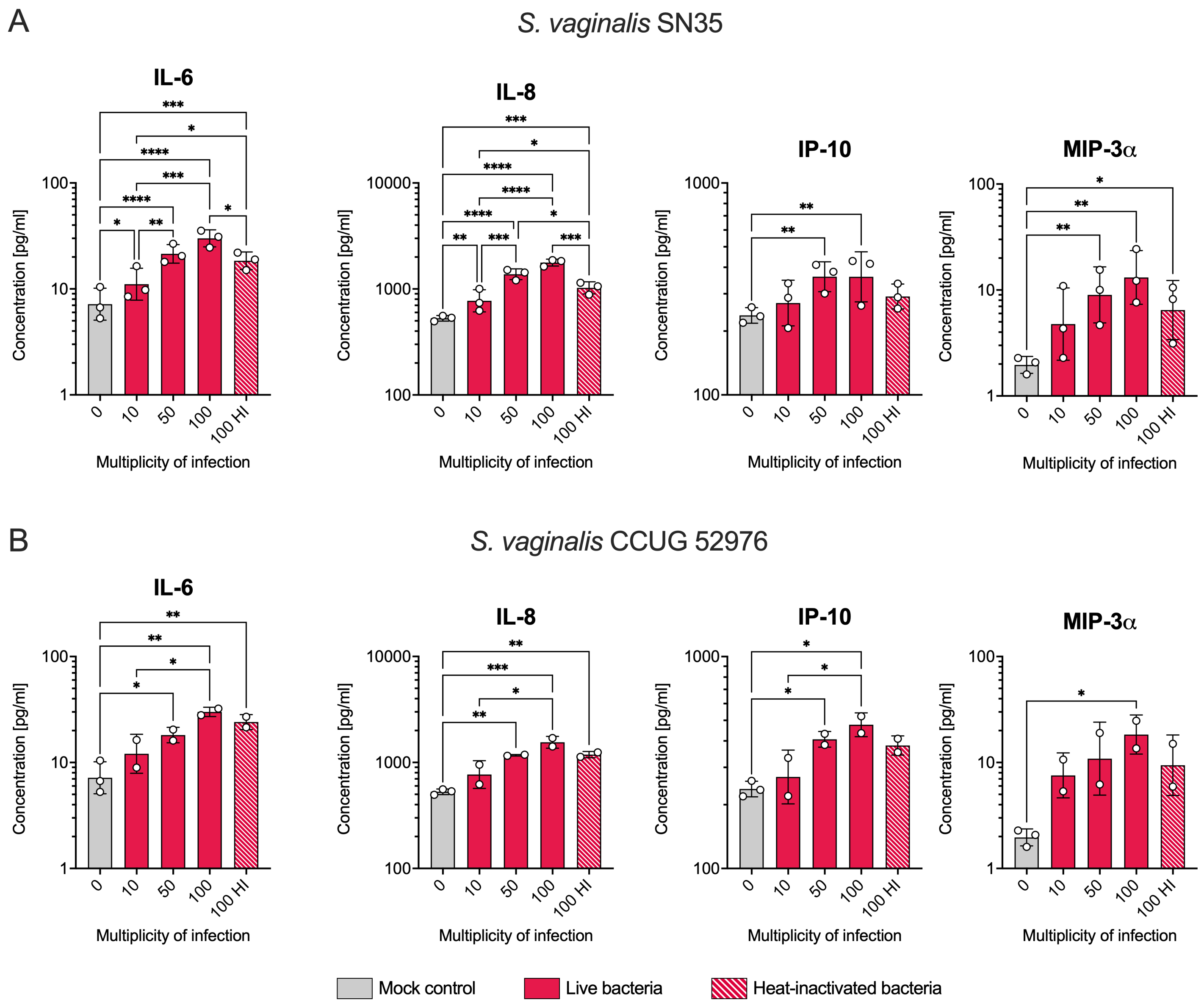

Supplementary Figure S5. Secretion of key immune mediators in a human 3D cervical epithelial cell model following infection with *S. vaginalis* at various multiplicities of infections (MOIs).

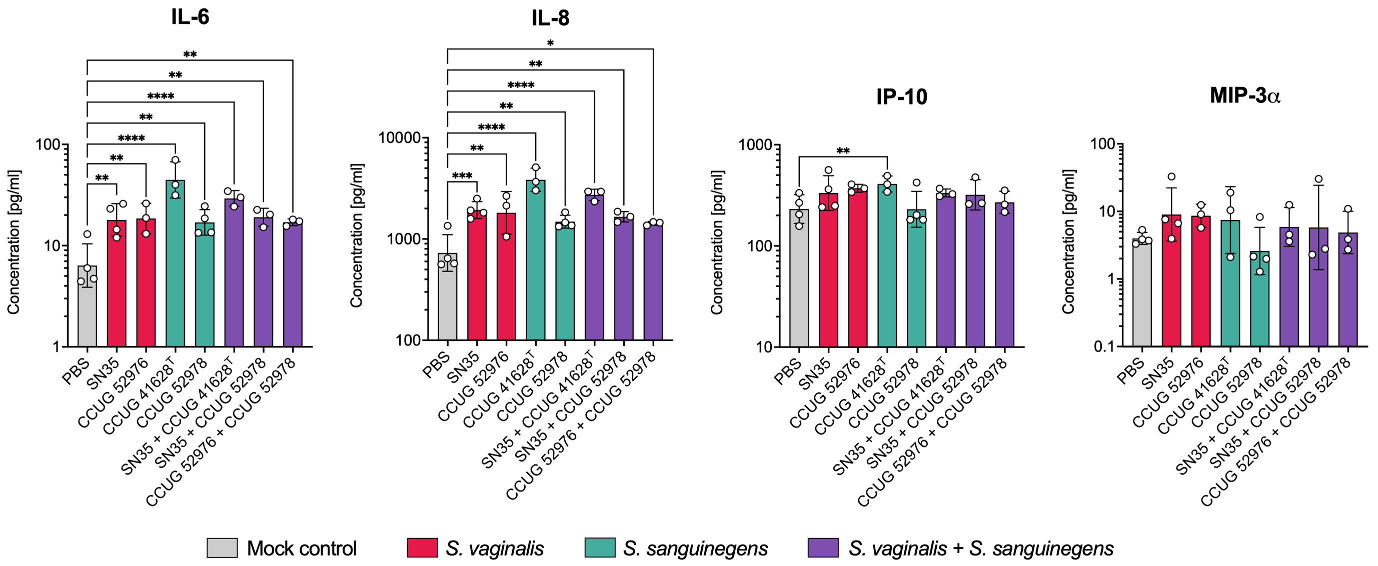

Supplementary Figure S6. Secretion of key immune mediators in a human 3D cervical epithelial cell model following dual-species infections (co-infections with *S. vaginalis* and *S. sanguinegens*) compared to single-species infections.
